# A Novel pH-Regulated, Unusual 603 bp Overlapping Protein Coding Gene *pop* Is Encoded Antisense to *ompA* in *Escherichia coli* O157:H7 (EHEC)

**DOI:** 10.3389/fmicb.2020.00377

**Published:** 2020-03-20

**Authors:** Barbara Zehentner, Zachary Ardern, Michaela Kreitmeier, Siegfried Scherer, Klaus Neuhaus

**Affiliations:** ^1^Chair for Microbial Ecology, Technical University of Munich, Freising, Germany; ^2^ZIEL – Institute for Food & Health, Technical University of Munich, Freising, Germany; ^3^Core Facility Microbiome, ZIEL – Institute for Food & Health, Technical University of Munich, Freising, Germany

**Keywords:** overlapping gene, EHEC O157:H7, pH, overexpression phenotypes, protein, ribosomal profiling

## Abstract

Antisense transcription is well known in bacteria. However, translation of antisense RNAs is typically not considered, as the implied overlapping coding at a DNA locus is assumed to be highly improbable. Therefore, such overlapping genes are systematically excluded in prokaryotic genome annotation. Here we report an exceptional 603 bp long open reading frame completely embedded in antisense to the gene of the outer membrane protein *ompA*. An active σ^70^ promoter, transcription start site (TSS), Shine-Dalgarno motif and rho-independent terminator were experimentally validated, providing evidence that this open reading frame has all the structural features of a functional gene. Furthermore, ribosomal profiling revealed translation of the mRNA, the protein was detected in Western blots and a pH-dependent phenotype conferred by the protein was shown in competitive overexpression growth experiments of a translationally arrested mutant *versus* wild type. We designate this novel gene *pop* (pH-regulated overlapping protein-coding gene), thus adding another example to the growing list of overlapping, protein coding genes in bacteria.

## Introduction

Due to the nature of the genetic triplet code, six reading frames exist on the two strands of a DNA molecule. Two genes encoded by two different reading frames (ORFs) at the same DNA locus are termed “non-trivially overlapping genes” (OLGs) if the area of sequence overlap is substantial (at least 90 base pairs) and both reading frames encode a protein. Such overlapping genes were discovered in bacteriophage ϕX174 by Barrell et al. as early as 1976 (Barrell et al., 1976). Today, the existence of protein coding OLGs is accepted in viruses, although the evolutionary pressures behind the development of gene overlaps are still debated. Theories about size constraint of the genome in the viral capsid, gene novelty, and evolutionary exploration have been discussed (Chirico et al., 2010; Brandes and Linial, 2016).

In contrast, most overlaps reported in bacterial genomes are very short; the majority being only 1 or 4 bp in same-strand orientation, and we term these trivially overlapping genes. Such very small overlaps seem to increase fitness (e.g., Saha et al., 2016) which might be explained by the translational coupling of expression of the overlapping genes. Due to requiring only a small-scale slippage of the ribosome, mediated by the short overlap, translation is faster and highly efficient in contrast to the conventional translation process which includes dissociation of the ribosome after translation of the upstream gene and time consuming re-association to the downstream ORF of the mRNA (Johnson and Chisholm, 2004).

Very little work has been devoted to the exploration of long overlapping reading frames in prokaryotes, where one ORF is embedded completely in the other ORF (Rogozin et al., 2002; Ellis and Brown, 2003). As bacterial genomes are typically much larger than those of viruses, the original hypothesis suggesting a selection pressure associated with the evolution of overlapping genes in viruses due to an increase of the coding capacity in size-restricted genomes (Normark et al., 1983) has been assumed to be invalid for prokaryotes. In line with this assumption, overlapping genes are systematically excluded in prokaryotic genome annotations (e.g., Warren et al., 2010), which is certainly one reason for the lack of knowledge about such amazing gene constructs in bacteria. Nevertheless, statistical analysis of bacterial genomes has shown that ORFs overlapping annotated genes in alternative reading frames are longer than expected, leading to the hypothesis of a potential selection pressure due to overlapping protein-coding genes (Mir et al., 2012). Besides this, functionality of at least a few non-trivially overlapping genes has been demonstrated (e.g., Behrens et al., 2002; Balabanov et al., 2012).

It is assumed that overlapping genes originated by overprinting of existing, annotated genes (Sabath et al., 2012) and may constitute an evolutionarily young part of the functional genome of bacteria (Fellner et al., 2014, 2015). In contrast to older genes with highly conserved and essential functions, young overlapping genes appear to have weak expression (Donoghue et al., 2011) and their protein functions are suggested to be not essential (Chen et al., 2012). Therefore, the task of functionally characterizing OLGs is challenging. In order to capture weak and condition-specific phenotypic effects caused by the weak expression of non-essential overlapping genes, sensitive methods are necessary (Deutschbauer et al., 2014).

We study non-trivially overlapping genes in the human pathogenic bacterium *Escherichia coli* O157:H7 (EHEC). Its genome is well characterized, especially with respect to virulence and the associated diseases like enterocolitis, diarrhea, and hemolytic uremic syndrome (Lim et al., 2010; Stevens and Frankel, 2014; Betz et al., 2016). Nevertheless, the coding capacity of EHEC’s genome is likely to be significantly underestimated, both regarding short intergenic genes (Neuhaus et al., 2016; Hücker et al., 2017) and non-trivially overlapping genes (Hücker et al., 2018a, b; Vanderhaeghen et al., 2018). Additionally, using a variety of different next generation sequencing based methods (e.g., RNAseq, Cappable-seq, ribosome profiling) evidence for widespread antisense transcription has accumulated (Conway et al., 2014). In particular, ribosome profiling has been shown to be a powerful technique to investigate the translated part of an organisms’ transcriptome with high precision, through deep sequencing of ribosome-protected mRNA fragments (Ingolia et al., 2009; Hwang and Buskirk, 2016; Nakahigashi et al., 2016). Furthermore, variations of this method were developed to resolve specific features of translation, such as alternative translation initiation sites, translational pausing or translation termination (Woolstenhulme et al., 2015; Baggett et al., 2017; Meydan et al., 2019). Based on such techniques, surprising additional complexity of the bacterial translatome has been uncovered. In particular, findings of putatively translated antisense RNAs could be very significant with respect to overlapping genes (Meydan et al., 2019). Nevertheless, the specificity of the signals found in all NGS experiments needs to be assessed and differentiated from a potentially pervasive background translation, i.e., undirected binding of ribosomes to RNAs (Ingolia et al., 2014). It was reported that pervasive translation initiation sites in bacteria predominantly lead to short translation products with an uncertain functionality status (Smith et al., 2019). However, the metabolic cost of pervasive translation would be high and cells should be driven to minimize such costly side reactions. To gather further evidence for an overlapping coding potential, individual overlapping genes have to be characterized in detail. Such research is in its infancy in bacteria.

Here, we report on a functional analysis of the unusually long, non-trivially overlapping gene *pop* from *E. coli* O157:H7 strain EDL933, which is fully embedded in antisense to the annotated gene of the outer membrane protein *ompA.* OmpA is highly conserved among proteobacteria and represents the major outer membrane protein in *E. coli* with about 100,000 copies per cell (Koebnik et al., 2000). Extensive studies led to the discovery of the β-barrel structure of OmpA (Vogel and Jähnig, 1986) as well as diverse functions of this protein, such as a porin function (Arora et al., 2001) and a local cell wall stabilizing action through interaction of OmpA with TolR (Boags et al., 2019).

## Materials and Methods

### Oligonucleotides, Bacterial Strains, and Plasmids

All oligonucleotides, bacterial strains and plasmids used or created in this study are listed in Supplementary Table S1.

### Media, Media Supplements, and Culture Conditions

All *E. coli* strains were cultivated in LB (10 g/L tryptone, 5 g/L yeast extract, 5 g/L NaCl) at 37°C, if not stated otherwise. If necessary, medium was supplemented with additives or stressors (see Supplementary Table S2).

### Cloning Techniques

Desired sequences were amplified from genomic DNA of *E. coli* O157:H7 EDL933 in a PCR [Q5 polymerase, New England Biolabs (NEB), Ipswich, MA, United States] using different primer pairs. PCR fragments were digested with appropriate restriction enzymes (Thermo Fisher Scientific, Waltham, MA, United States) and ligated in the multiple cloning sites of application specific vectors with T4 DNA ligase (Thermo Fisher Scientific). Vector constructs were transformed in *E. coli* Top10 cells and plated on LB with required antibiotics. Plasmids were isolated (GenElute Plasmid Miniprep Kit, Sigma Aldrich, St. Louis, MO, United States) and sequenced with suitable primers (Eurofins Genomics, Ebersberg, Germany) to verify the sequence.

### Creation of Translationally Arrested Knock-Out Mutants

The genomic knock-outs *E. coli* O157:H7 EDL933 Δ*pop* and *E. coli* O157:H7 EDL933 Δ*pop* v2 were produced for subsequent competitive growth experiments. The method was adapted from Fellner et al. (2014). Mutation fragments were amplified with primer pairs 1 + 6 and 2 + 5 for the knock-out Δ*pop.* For the knock-out Δ*pop* v2, primer pairs 3 + 7 and 4 + 5 were used. The fragments gained were used in the subsequent overlap extension PCR with primers 5 + 6 or 5 + 7, respectively. The resulting mutation cassettes, Δ*pop* and Δ*pop* v2, were cloned in the plasmid pMRS101 (Sarker and Cornelis, 1997) using *Apa*I/*Spe*I and *Apa*I/*Xba*I, respectively (selection with ampicillin). The plasmids pMRS101+Δ*pop* and pMRS101+Δ*pop* v2 were isolated and sequenced with primers 7 and 8, respectively. The following steps were performed for both plasmids: A restriction digest with *Not*I was conducted to remove the high copy ori. The plasmid was re-ligated to the π-protein dependent, low copy plasmid pKNG101+x (x denotes either insert Δ*pop* or Δ*pop* v2*)*, whose maintenance relies either on cells expressing the *pir* gene, which enables replication, or on integration of the plasmid via homologous recombination – in case the cell does not express the *pir* gene (Kaniga et al., 1991). Plasmid propagation was performed in *E. coli* CC118λpir (selection with streptomycin). The conjugation strain *E. coli* SM10λpir was transformed with pKNG101+x. Overnight cultures (500 μl) of *E. coli* SM10λpir + pKNG101+x and *E. coli* O157:H7 EDL933 + pSLTS (selection marker ampicillin, temperature sensitive ori) were mixed and cultivated on LB plates (24 h, 30°C) for conjugation and integration of the plasmid into the genome of EHEC through homologous recombination. Conjugated EHEC cells were transferred on LB/ampicillin/streptomycin plates and selectively cultivated (24 h, 30°C). Correct insertion of the plasmid was confirmed by a PCR using primers 8 + 12 for pKNG101+Δ*pop* or 10 + 12 for pKNG101+Δ*pop* v2. A double-resistant strain was used for loop-out of the mutation plasmid. For this, conjugated EHEC + pSLTS was cultivated in LB at 30°C at 150 rpm until an optical density of OD_600_ = 0.5 and counter-selected on sucrose agar (modified LB without NaCl supplemented with sucrose) containing 0.02% arabinose to induce the λ red recombination system on pSLTS. Cells with integrated pKNG101+x express the enzyme levansucrase, encoded by the gene *sacB*, which catalyzes the hydrolysis of sucrose and synthesis of levans. It is proposed that these toxic fructose polymers accumulate in the periplasm of Gram-negative bacteria leading to cell death (Reyrat et al., 1998). Therefore, only sucrose-resistant cells, achieving the second recombination step, have lost the plasmid with its streptomycin resistance. PCR fragments of streptomycin sensitive clones produced with primers 8 + 9 and 10 + 11 for EHEC Δ*pop* and EHEC Δ*pop* v2, respectively, were sequenced to verify integration of the desired mutations into the chromosome. *E. coli* O157:H7 EDL933 Δ*pop* and *E. coli* O157:H7 EDL933 Δ*pop* v2 were cultivated at 37°C to cure the cells from the plasmid pSLTS.

### Cloning of pBAD+*pop* and pBAD+Δ*pop* for Overexpression Phenotyping

For overexpression competitive growth testing, plasmids pBAD+*pop* and pBAD+Δ*pop* were constructed. For the former construct, primers 14 + 15 were used. The latter construct was created similarly to the mutation cassette described in the previous section (i.e., primers for the mutation fragments are 1 + 14 and 2 + 15; primers for the mutation cassette are 14 + 15). Both PCR fragments, either wild type or mutant, were cloned in the *Nco*I and *Pst*I sites of pBAD/myc-HisC and plasmids were sequenced with primers 16 + 17. Each of the plasmids was transformed in wild type *E. coli* O157:H7 EDL933 for subsequent competitive growth assays.

### Competitive Growth Assays

For competitive growth, overnight cultures of EHEC transformants containing pBAD+*pop* or pBAD+Δ*pop* were diluted to OD_600_ = 1 and mixed in equal amounts. Plasmids were isolated from the bacteria mixture and used as time point zero reference. One hundred microliters of a 1:300 dilution of the initial 1:1 bacteria mixture was used to inoculate 10 ml culture medium with appropriate additives (for working concentration of chemicals see Supplementary Table S2; selection marker ampicillin for plasmid maintenance). Overexpression of *pop* and Δ*pop* cloned on pBAD was induced with L-arabinose (0.02%) added at the two time points *t*_0_ = 0 h and *t*_1_ = 6.5 h. Plasmids were isolated after *t*_2_ = 22 h and sequenced with primer 16. The competitive index, based on t_0_ of the mixture *pop* wild type and *pop* mutant expressing cells, was calculated. For this, the peak heights (fluorescence signals in Sanger sequencing) of mutated and wild type base at the mutated position were measured. The CI values were calculated according to this formula: CI = (*Mt*_*x*_/*Wt*_*x*_)/(*Mt*_*t*__0_/*Wt*_*t*__0_) with *Wt* and *Mt* the peak heights of wild type and mutant plasmid, respectively, in stress condition or reference condition *t*_0_. Mean values and standard deviations of at least three biological replicates were calculated. Significance of a possible growth phenotype was tested with a paired *t*-test between CI values of the time point t_0_ reference and the cultured samples (*p*-value ≤ 0.05).

Competitive growth of wild type EHEC and translationally arrested mutants *E. coli* O157:H7 EDL933 Δ*pop* or *E. coli* O157:H7 EDL933 Δ*pop* v2 was conducted and evaluated as described above with some exceptions: no selection marker was used; no protein expression was induced; cells were harvested after *t*_*x*_ = 18 h; peak heights were determined in t_0_ and cultured samples by sequencing PCR products amplified from cell lysates with primers 8 + 9 or 10 + 11 for Δ*pop* or Δ*pop* v2 used in competitive growth, respectively (primer for sequencing: 8 or 11).

### Copy Number Estimation

Overnight culture of *E. coli* O157:H7 EDL933 with either pBAD+*pop* (*pop* sample) or pBAD+Δ*pop* (Δ*pop* sample) were diluted to OD_600_ = 1. Diluted cultures (1:300) were used to inoculate 10 ml LB, LB + malic acid or LB + bicine (for working concentration of chemicals see Supplementary Table S2; selection marker ampicillin for plasmid maintenance). Transcripts of *pop* and Δ*pop* were induced as described for the competitive growth assay. DNA (genomic and plasmid) was isolated after growth of 22 h using phenol/chloroform/isoamyl alcohol (Carl Roth, Karlsruhe, Germany). For this, cultured cells were pelleted and resuspended in 700 μl Tris/EDTA (pH 8) and disrupted with bead beating (0.1 mm zirconia beads) using a FastPrep (three-times at 6.5 ms^–1^ for 45 s, rest 5 min on ice between the runs). The cell debris was removed after centrifugation (5 min, 16.000 × *g*, 4°C). Nucleic acids in the supernatant were extracted with 1 Vol phenol/chloroform/isoamyl alcohol twice (vigorously shaking, 5 min, 16.000 × *g*, 4°C) and precipitated using 2 Vol 100% EtOH and 0.1 Vol 5M NaOAc at −20°C for at least 30 min. After centrifugation (10 min, 16.000 × *g*, 4°C), the cell pellet was washed twice with 1 ml 70% EtOH (incubation 5 min at room temperature, centrifugation 5 min, 16.000 × *g*, 4°C). The dried pellet was rehydrated with an appropriate amount of water. RNA was digested using 0.1 Vol of RNase A (Thermo Fisher Scientific) and DNA was recovered by phenol/chloroform/isoamyl alcohol isolation as before.

Genomic and plasmid DNA was relatively quantified in biological and technical triplicates by qPCR using a genomic specific primer pair amplifying a 105 bp long fragment of the siroheme synthase gene *cysG* (primer 34 + 35, Zhou et al., 2011) and plasmid specific primers amplifying a 101 bp long fragment of the β-lactamase gene *bla* (primer 36 + 37, Roschanski et al., 2014). DNA samples were used at a concentration of 100 ng/μl. Amplification cycle differences were calculated for each of the culture conditions [ΔCq(*cysG-bla*)] for *pop* and Δ*pop* DNA samples. The ratio of condition specific ΔCq values for *pop*/Δ*pop* samples was calculated to estimate the deviation of copy numbers in cells overexpressing either of the plasmids. Statistically significant differences of copy number ratios between *t*_0_ and each cultured sample was tested for with a paired two sample *t*-test (*p*-values ≤ 0.05).

### Construction of an Overexpression Plasmid and Western Blot

The plasmid pBAD/myc-HisC, which codes for the peptide tags myc and 6xHis, was modified to obtain the overexpression plasmid pBAD/SPA with the SPA-tag instead (sequential peptide affinity tag, dual epitope tag, consists of calmodulin binding peptide and 3xFLAG-tag separated by a TEV protease cleavage site, Zeghouf et al., 2004). For this, primers 19 + 20 were annealed (heating at 90°C, slow cooling) and completed in a PCR where primers 21 + 22 were added after 5 cycles to amplify the fragment. This PCR product was cloned into pBAD/myc-HisC using *Sal*I and *Hin*dIII restriction enzymes. This resulted in an excision of the myc-epitope and in-frame insertion of the SPA-tag. The sequence of *pop* was cloned next after amplification with primers 14 + 18 in the *Nco*I and *Hin*dIII sites of pBAD/SPA. The plasmid pBAD/SPA+*pop* was sequenced with primers 16 and 17 for verification and transformed into *E. coli* O157:H7 EDL933.

Overexpression was performed in LB medium and bicine-buffered LB medium. Cells were cultivated and protein production was induced with 0.002% arabinose when an optical density of OD_600_ = 0.3 was reached. Cells were harvested right before induction (uninduced control) and at time points 0.5, 1, 1.5, 2, 2.5, 3, and 4 h after induction. The cell volume harvested was adjusted to achieve the same OD_600_ for all samples regarding uninduced cells (OD_600_ = 0.3). Whole cell lysates were prepared by adding 50 μl SDS sample buffer (2% SDS, 2% β-mercaptoethanol, 40% glycerin, 0.04% Coomassie blue G250, 200 mM tris/HCl; pH 6.8) and heating at 95°C for 10 min. Proteins in 10 μl of the lysates were separated on a 16% tricine gel prepared according to Schägger (2006), and detected afterward in a Western blot. For this purpose, proteins were blotted semidry (12 V, 20 min) on a PVDF membrane (PSQ membrane, 0.2 μm, Merck Millipore, Burlington, Massachusetts, United States). After incubating the membrane 5 min in 3% TCA, it was blocked with non-fat dried milk at 4°C. After three washing steps (TBS-T), the membrane was incubated in a 1:1000 dilution of ANTI-FLAG^®^ M2-Alkaline Phosphatase antibody (Sigma Aldrich), which binds the FLAG epitope of SPA-tagged proteins, in TBS-T. SPA tagged proteins were visualized with BCIP/NBT.

### Determination of Promoter Activity by a GFP Assay

The promoter sequence of *pop* was amplified with primers 23 + 24. The product was cloned N-terminally into the promoterless GFP-reporter plasmid pProbe-NT using restriction enzymes *Sal*I and *Eco*RI resulting in pProbe-NT+promoter-*pop.* The promoter sequence was verified by sequencing the plasmid with primer 25. The promoter activity was measured in *E. coli* Top10. For this, 10 ml LB with the appropriate additive (for working concentration of chemicals see Supplementary Table S2; selection marker kanamycin) was inoculated 1:100 with overnight cultures of *E. coli* Top10, *E. coli* Top10 + pProbe-NT, and *E. coli* Top10 + pProbe-NT+promoter-*pop* and cultivated up to OD_600_ = 0.6. An appropriate number of cells were harvested, washed once and afterward resuspended in 1xPBS. Fluorescence of 200 μl cell suspension was measured in four technical replicates (Victor3, Perkin Elmer, excitation 485 nm, emission 535 nm, measuring time 1 s). Self-fluorescence of cells was subtracted. Mean values and standard deviation of three independent biological replicates were calculated. Statistically significant differences in the fluorescence of promoter construct and empty plasmid or between promoter constructs in different growth conditions were determined using the Welch two sample *t*-test (*p*-value ≤ 0.05).

### RNA Isolation

RNA was isolated from exponentially grown EHEC cultures (OD_600_ = 0.3 in LB, LB + L-malic acid, LB + bicine) using Trizol Reagent (Thermo Fisher Scientific). Cell pellets were resuspended in 600 μl cooled Trizol and disrupted with bead beating (0.1 mm zirconia beads) using a FastPrep (3-times at 6.5 ms^–1^ for 45 s, rest 5 min on ice between the runs). Cooled chloroform (120 μl) was added, mixed vigorously and incubated 5 min at room temperature. Phases were separated by centrifugation for 15 min (4°C, 12000 × *g*) and total RNA in the aqueous upper phase was precipitated with isopropanol, NaOAc and glycogen (690, 27, and 1 μl, respectively) at −20°C for 1 h. RNA was pelleted by centrifugation for 10 min and washed twice with 80% ethanol. Air-dried RNA was dissolved in an appropriate volume of RNase-free H_2_O.

### DNase Digestion

DNA in RNA samples was digested with Turbo DNase (Thermo Fisher Scientific) according to the manufacturer’s instructions. The reaction was stopped with 15 mM EDTA and heating for 10 min at 75°C. Digested RNA was precipitated with isopropanol, NaOAc and glycogen (690, 27, and 1 μl, respectively) at −20°C overnight. After centrifugation (20 min, 12000 × *g*), the pellet was washed once with 80% ethanol. Air-dried RNA was dissolved in an appropriate volume of RNase-free H_2_O. Successful DNA depletion was verified with a standard PCR using *Taq*-polymerase (NEB) and primers 26 + 27 binding to the 16S rRNA genes.

### cDNA Synthesis and RT-PCR

DNA-depleted total RNA (500 ng) was used for cDNA synthesis with SuperScript III reverse transcriptase (Invitrogen, Thermo Fisher Scientific) according to the manufacturer using 50 pmol random nonamer primer for 16S rRNA reverse transcription (Sigma Aldrich) or 10 pmol gene specific primers for *pop* reverse transcription as indicated. SUPERase In RNase Inhibitor (20 U/μl, Invitrogen) was added as well. “No RT” controls contained all components apart from the reverse transcriptase. For RT-PCR, 1 μl of the cDNA sample was used in a standard PCR using *Taq*-polymerase (NEB) with 20 cycles for product amplification using the primer pairs indicated. Binding of primers was verified in a PCR with genomic DNA as template (not shown).

### Quantitative PCR (qPCR)

Relative quantification of *pop* RNA and 16S rRNA based on cDNA (reverse transcribed with primer 8 and random nonamer primer, respectively) was conducted by qPCR using the SYBR Select Master Mix (Applied Biosystems). The reactions contained 12.5 μl master mix, 0.5 μl of forward and reverse primer (50 μM) and 1 μl cDNA at a total volume of 25 μl. Amplification of *pop* and 16S rRNA was performed with primers 8 + 9 and 26 + 27, respectively. The reaction conditions were as follows: 95°C (5 min, initial denaturation), 40 cycles of denaturation, annealing and elongation at 95°C (15 s), 61°C (30 s), and 72°C (30 s). Finally, a melting curve was acquired for quality control of the amplification products (61°C to 95°C in 0.5°C steps for 5 s). qPCR was performed in three biological replicates in each condition (LB, LB + L-malic acid, and LB + bicine) with three technical replicates for every sample. A no-RT control was included for all samples to verify specificity of the amplification from cDNA (e.g., exclude DNA contamination). *pop* mRNA was quantified with the ΔΔCq method using 16S rRNA as reference (Pfaffl, 2001). Statistical significance was calculated by means of a one-tailed Welch two sample *t*-test (*p*-value ≤ 0.05).

### Bioinformatic Analysis

#### Promoter Determination

The programs BPROM (Solovyev and Salamov, 2011) and bTSSfinder (Shahmuradov et al., 2017) were used to determine the promoter of *pop*. The input sequence for BPROM was 100 bp long and started 65 bp upstream of the identified TSS. The input for bTSSfinder needed to be longer; it spans 300 bp and starts 197 bp upstream of the TSS. BPROM specifies the promoter strength as a linear discriminant function (LDF) and a sequence with LDF = 0.2 indicates a promoter with 80% accuracy and specificity. bTSSfinder calculates scores based on position weight matrices for different sigma factors and accepts promoters greater than the scoring thresholds (0.06 for σ^70^).

#### Terminator Analysis

The program FindTerm (Solovyev and Salamov, 2011) was used to analyze 900 bp downstream of *ompA* for a rho-independent terminator (threshold −3). The 120 bp long terminator identified was split consecutively into 30 bp segments and all 91 sequences were folded with Mfold (Zuker, 2003) to identify the stem loop structure.

#### Shine-Dalgarno Sequence Identification

Presence of a Shine-Dalgarno sequence in the region 30 bp upstream of the start codon was analyzed according to Ma et al. (2002). A minimum of Δ*G*° = −2.9 kcal/mol is required for detection of a ribosome binding site.

#### Gene Prediction

Genome sequences and assembly data of *E. coli* O157:H7 EDL933 (Accession number CP008957), *Shigella dysenteriae* str. ATCC 13313 (Accession number CP026774), *Klebsiella pneumoniae* subsp. *pneumoniae* str. ATCC 13883 (BioProject PRJNA261239), and *Enterobacter cloacae* subsp. *cloacae* str. ATCC 13047 (Accession number CP001918) were downloaded from NCBI. Gene prediction was performed with Prodigal v2.60 (Hyatt et al., 2010) with default settings.

#### Ribosomal Profiling Analysis

Ribosome profiling data of *E. coli* O157:H7 EDL933 (Neuhaus et al., 2017), samples in LB for two biological replicates, SRR5266618, SRR5266620), *E. coli* O157:H7 Sakai [Hücker et al. (2017), sample in LB, SRR5874484; files for the two separate biological replicates were kindly provided by Sarah Hücker] and *E. coli* MG1655 [Wang et al. (2015), samples in LB for two biological replicates; ERR618775, ERR618771] were downloaded from NCBI. Data for *E. coli* LF82 (GenBank accession: NC_011993) was produced in our lab according to the methods of Hücker et al. (2017) in Schaedler broth medium (anaerobic cultivation). Data evaluation was conducted as following: Adapters were trimmed with cutadapt (Martin, 2011) with a minimum quality score of 10 (q 10) and minimum length of 12 nucleotides (m 12). The trimmed reads were subsequently aligned to the reference chromosome using bowtie2 (Langmead and Salzberg, 2012) in local alignment mode, with zero mismatches (N 0) and a seed length of 19 (L 19). Reads overlapping ribosomal and tRNAs were removed using bedtools (Quinlan and Hall, 2010). Read counts, RPKMs, and coverage were then calculated with respect to the filtered BAM files, using bedtools and a custom bash script.

Stalled-ribosome profiling data from the *E. coli* strain BL21 was obtained from Meydan et al. (2019). The adapter sequence was predicted using DNApi.py (Tsuji and Weng, 2016), and adapter trimming, alignment, and removal of rRNAs and tRNAs was conducted as described above. The positions of all reads mapped to the forward strand were obtained using SAMtools (Li et al., 2009) and the “bamtobed” tool from BamTools (Barnett et al., 2011). Reads with predicted ribosomal *p*-sites within 30 nucleotides in each direction of an annotated forward-strand gene start codon (“start region”) were extracted. Weakly expressed annotated genes with no single position (peak) represented by three or more reads, and also with at least four reads situated within the start region, were found using a custom bash script, as a positive control for weak gene expression.

## Results

### Localization of *pop* in the Context of the EHEC Genome and Its Expression

**FIGURE 1:**
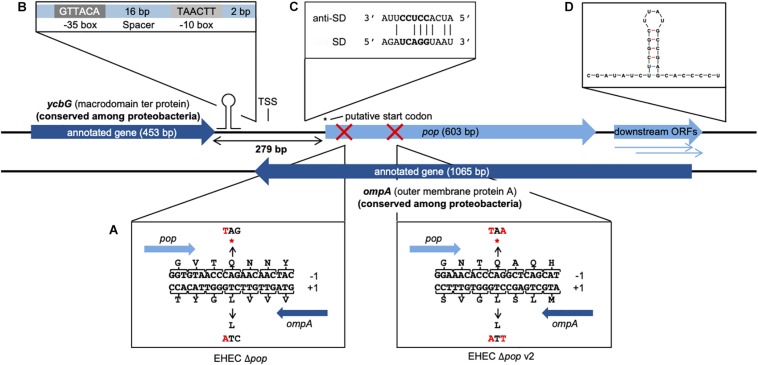
Genomic organization and operon structure of *pop*. *pop* (603 bp) is located downstream of the annotated gene *ycbG* and completely embedded antisense in the sequence of *ompA*. Downstream of *pop* exist two smaller overlapping open reading frames, which overlap *ompA* almost completely and are referred to as downstream ORFs. A transcription start site (TSS) was experimentally identified in the intergenic region of *pop* and *ycbG*. The full genomic sequence of *pop* is given in Supplementary Figure S1. **(A)** Design of translationally arrested mutants of *pop*. The overlapping ORF *pop* is located in reading frame −1 with respect to *ompA*. Genomic mutants for phenotypic characterization contained a single base substitution C → T at genome position 1236083 for EHEC Δ*pop* or C → T and G → A at genome positions 1236302 and 1236304 for EHEC Δ*pop* v2 (indicated with red crosses), leading to stop codons (*) in *pop* and synonymous changes in *ompA*. **(B)** Promoter sequence. The sequences of the −10 box and −35 box as well as the length of the spacer between the conserved boxes and the distance to the TSS are shown. **(C)** Alignment of Shine-Dalgarno (SD) sequence of *pop* and anti-Shine-Dalgarno (anti-SD) sequence. The predicted SD sequence (Δ*G*° = −3.6 kcal/mol) upstream of the putative start codon is aligned to the consensus of the anti-SD sequence of the 16S rRNA in the 30S ribosomal subunit (Ma et al., 2002). The core of the ribosome binding site is displayed in bold letters. **(D)** Secondary structure of the first 40 bp of the predicted terminator. The folding was conducted with Mfold and the structure has a final energy of ΔG = −8.6 kcal/mol.

**FIGURE 2:**
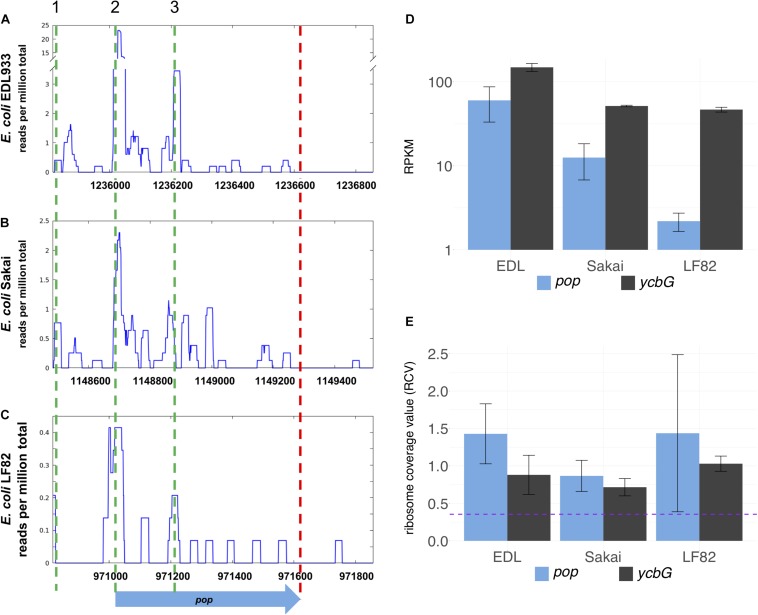
Inter-strain comparison of *pop* translation. Alignment of sequence and ribosomal profiling reads of *pop* and its homologs across pathogenic *E. coli* strains O157:H7 EDL933 **(A)**, O157:H7 Sakai **(B)**, and LF82 **(C)**. Graphs show normalized sequencing reads (RPKM, reads per kilobase per million mapped reads) of ribosome profiling experiments in LB medium **(A,B)** or Schaedler broth **(C)**; the sum signal of two biological replicates is visualized. Three putative start codons are indicated with green dashed lines in region 1 (TTG), 2 (CTG), and 3 (GTG). The stop codon is indicated by a red dashed line. **(D)** Averaged RPKM values of translation and **(E)** ribosomal coverage values (RCV) of overlapping gene *pop* and the upstream annotated gene *ycbG* of three *E. coli* strains. Purple dashed line in panel **(E)**: threshold for translated ORFs, RCV = 0.35. Error bars indicate both underlying values used for calculations.

The overlapping gene *pop* from *E. coli* O157:H7 (EHEC) EDL933 probably starts at genome position 1236020 (coordinates following the genome annotation of Latif et al. (2014), GenBank accession CP008957) and has a length of 603 bp (Figure 1 and Supplementary Figure S1). It is completely embedded in antisense to the coding sequence of the annotated, highly conserved outer membrane protein gene *ompA* (1065 bp). *pop* is located in frame −1 with respect to *ompA* (Figure 1A). Ribosome profiling of EHEC EDL933 revealed clear evidence of translation of this OLG in LB medium, which is reproducible across biological replicates (Figure 2A and Supplementary Table S3) and EHEC strains (Figures 2A–C, see below). Expression of *ompA* is about 150 times higher than *pop*, which is not surprising since OmpA is one of the most highly expressed proteins in *E. coli* (Ortiz-Suarez et al., 2016). The annotated gene *ycbG* (453 bp), encoding a macrodomain ter protein, is located upstream of *pop.* RPKM (reads per kilobase per million mapped reads) values of *ycbG* are on average three times higher than values of *pop* (Supplementary Table S3 and Figure 2D). However, the RPKM of *pop* in ribosome profiling of EDL933 (i.e., RPKM ≈ 60) is at the same order of magnitude as the median RPKM of all annotated genes with an RPKM of at least 10 (RPKM = 70 and RPKM = 63 for ribosome profiling experiments SRR5266618 and SRR5266620, respectively), supporting genuine expression of *pop*. In addition to the level of protein expression given by the ribosome profiling RPKM value, the ribosome coverage value (RCV) describes the “translatability” of a particular gene’s messenger RNA, i.e., $\mathrm{RCV}=\frac{\mathrm{RPKM}\,\left(\mathrm{translatome}\right)}{\mathrm{RPKM}\,\left(\mathrm{transcriptome}\right)}$ (Hücker et al., 2017). For *pop*, the RCV is high, greater than 1 in a few instances. According to Neuhaus et al. (2017), transcripts with an RCV higher than 0.35 can be considered to be translated, while untranslated RNAs have a clearly lower RCV. Therefore, we propose that *pop* is translated in all pathogenic *E. coli* strains investigated. Notably, the RCV as measure of the translation of an mRNA into protein is on average higher for *pop* than for the annotated upstream gene *ycbG* (Figure 2E and Supplementary Table S3). The finding reinforces our hypothesis that the ribosome profiling signals found for the *pop* coding region are meaningful and this is clear evidence for translation of *pop*. Expression of *pop* was analyzed in three pathogenic *E. coli* strains (O157:H7 EDL933, O157:H7 Sakai, and LF82) and an *E. coli* K12 strain (MG1655; Figures 2A–C and Supplementary Table S3). Interestingly, *pop* is translated in EDL933, Sakai, and LF82, with highest values in EDL933, whereas it is neither transcribed nor translated in *E. coli* MG1655, indicated by low RPKM values and a low RCV.

The region between *ycbG* and *pop* contains the transcription start site (TSS) and a σ^70^ promoter (Figure 1B, details further below). Two downstream ORFs, which are arranged in frames −1 and −2 with respect to *ompA*, are a little over 200 bp long and mostly overlap with *ompA* (Figure 1). Despite a downstream rho-independent terminator (Figure 1D, details further below) neither of these ORFs appears to be transcribed or translated to a major degree (Supplementary Table S3) and, therefore, we designate the two ORFs in the following simply as downstream ORFs.

**FIGURE 3:**
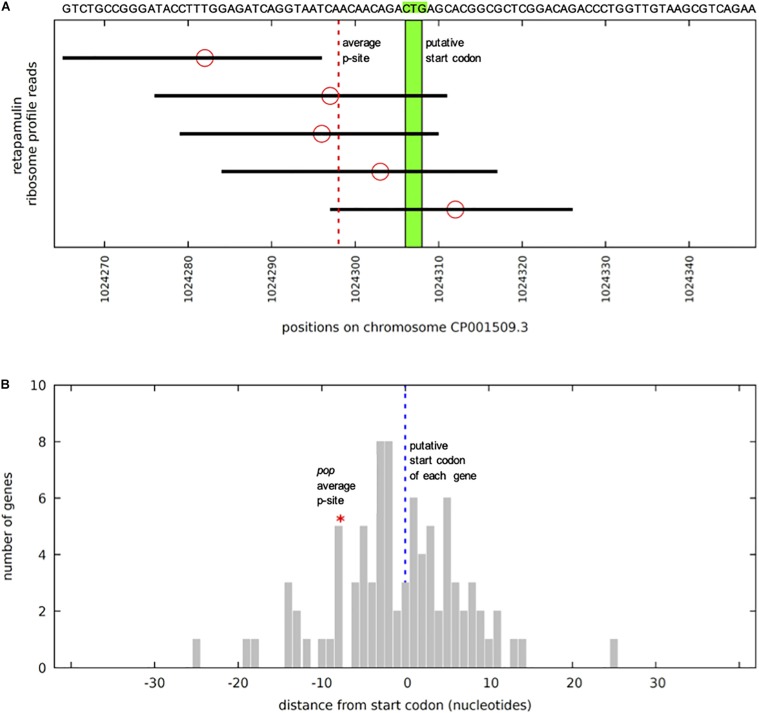
*pop* in stalled-ribosome profiling data of *E. coli* BL21. **(A)** Reads (black bars) antisense to *ompA* in the region of *pop* (sequence indicated above). The five reads situated on the forward strand antisense to *ompA* are shown, aligned to the BL21 chromosome CP001509.3. Ribosomal *p*-site locations (red circles) are predicted based on the method of Meydan et al. (2019) – counting 15 nucleotides from the 3′ end of the read. As there is no clear peak, instead the mean of all of the *p*-sites was calculated. The mean is shown with a dotted red line, pictured in relation to the putative start codon CTG in green. Significance of 5 reads was calculated based on the read distribution modeled as a binomial process. The total sequence space available is 3943267 nucleotides. If we conservatively assume a target size of 100 bp, this equates to a probability of success for a single trial of 100/3943267 = 0.0000254. We find 56812 reads antisense to annotated genes, which is the number of independent trials, if we generously assume as the null hypothesis that all are due to non-functional “background” translation events. With these parameters, the probability of obtaining five or more reads in our target region, i.e., *P*(*x* ≥ 5), is equal to 0.0159, therefore 5 reads are significant (*p*-value ≤ 0.05). **(B)** Average ribosomal *p*-site positions for 85 weakly expressed genes. Positions of average *p*-sites relative to annotated start codons (blue dotted line), as illustrated in panel **(A)**, are plotted for all 85 weakly expressed forward (+) strand annotated gene start regions. Weakly expressed is here defined as having at least four mapped reads within 30 nucleotides of the annotated start site, but no single position (peak) with three or more reads. The location of the average *p*-site for *pop* (red asterisk) lies within this distribution, indicating that the observed cluster of ribosome-stalled reads near the CTG site is informative. Thus, we assume that CTG might be the start codon for *pop*.

Upstream of the *pop*-ORF, we detected a Shine-Dalgarno sequence (ΔG° = −3.6 kcal/mol) and the rare start codon CTG nearby (position 1236020, Figure 1C, see also Supplementary Figure S1). Additional evidence for this CTG probably being the translation initiation site is found in recently published stalled-ribosome profiling data using the antibiotic retapamulin in the strain BL21 (Meydan et al., 2019). This antibiotic leads to an arrest of ribosomes starting biosynthesis in the region of translation initiation. Five reads are antisense to *ompA*, and all of these are clustered in the vicinity of the putative CTG start site of *pop* (Figure 3A). The read count observed would be unexpected if the cause was a random background translation event. A very conservative calculation of the binomial probability gives *P*(*x* ≥ 5) = 0.016 indicating non-random clustering of reads antisense to *ompA* at the *pop* start site (Figure 3A). A comparison to weakly expressed annotated genes (selection described in methods section) shows that the putative location of the *pop* translation initiation site is within the typical range for such genes (Figure 3B), and provides evidence locating the start codon within at most a few nucleotides of the predicted site. Similarly, we find that with pooled ribosome profiling data from EDL933, using the method of Meydan et al. (2019) to predict the ribosomal *p*-site as described in their methods section precisely identifies the start of the previously mentioned CTG codon (position 1236020) as a translation initiation site.

In summary, *pop* was identified as a translated open reading frame based on ribosome profiling experiments. In the following, we present further data supporting a protein-coding status for the gene as well as expression and functionality of this overlapping gene in the human pathogenic bacterium *E. coli* O157:H7 EDL933.

### Overexpression Phenotypes Indicate Functionality of *pop*

Competitive growth experiments were conducted to analyze the influence of *pop* on EHEC’s growth. For this purpose, the longest possible ORF of *pop* and the translationally arrested mutant ORF Δ*pop* were cloned in an overexpression plasmid under the control of an arabinose-inducible promoter with the optimal ribosome binding site of the plasmid (pBAD+*pop* and pBAD+Δ*pop*). The mutant plasmid differs in just one base from the wild type plasmid and this single base substitution introduces a stop codon in the overlapping gene (Figure 1A). It is assumed that these small alterations do not change the activity and function of expressed *pop* RNA possibly working as interfering ncRNA. This would indeed affect *ompA* RNA levels but for both plasmids equally. However, protein production from the *pop* gene is ceased in only pBAD+*pop*. Thus, any difference in growth after overexpression of either the intact or the mutated *pop*-ORF can be explained by the presence or absence of a protein (i.e., Pop) encoded by this OLG.

**FIGURE 4:**
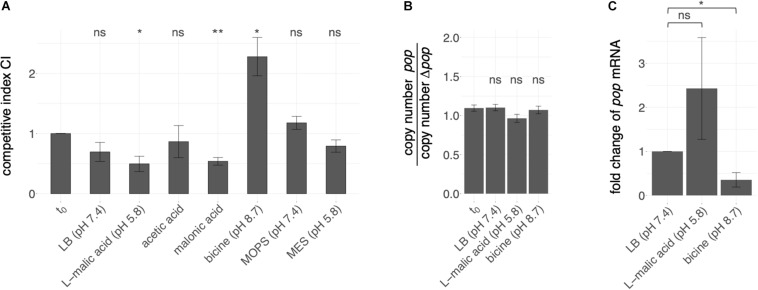
Effect of *pop* expression in various pH ranges. **(A)** Overexpression phenotypes of *pop* in competitive growth assays. Competitive growth of EHEC while overexpressing either intact (pBAD+*pop*) or translationally arrested *pop* (pBAD+Δ*pop*) was conducted in conditions as indicated, i.e., LB medium supplemented with an organic acid or a biological buffer. Mean competitive indices CI are given as the ratio of relative abundance of cells expressing mutant or wild type plasmid measured by peak heights, i.e., fluorescence intensities in sequencing electropherograms at the mutated positions, in different culture conditions regarding the input ratio t_0_. Error bars indicate standard deviations. Statistical significant differences of the CI (tested with paired *t*-test) before and after growth is indicated (* *p* ≤ 0.05; ** *p* ≤ 0.01; ns, not significant). **(B)** Copy number estimation for both plasmids. Genomic and plasmid DNA of EHEC transformants (pBAD+pop and pBAD+Δ*pop*) separately grown in the indicated growth conditions was relatively quantified by qPCR of a genome section (*cysG*-gene) and plasmid (*bla*-gene) specific gene segment. The mean ratios of quantification cycle differences [ΔCq(Cq(*cysG*)-Cq(*bla*)) = copy number] for the two transformants of three biological replicates are given. Error bars indicate standard deviations. No statistical significant ratio difference before and after growth was detected (tested with paired *t*-test; ns, not significant, *p*-value > 0.05) **(C)** *pop* expression measured by quantitative PCR (qPCR). Fold change of *pop* mRNA has been calculated based on ΔCq (difference in cycles of quantification) values of *pop* mRNA and 16S rRNA of EHEC grown to early exponential phase (OD_600_ = 0.3) in LB, LB + L-malic acid or bicine-buffered LB medium. Mean values and standard deviations of three biological replicates are shown. Statistical significance was tested with one-tailed Welch two sample *t*-test (* *p* ≤ 0.05; ns, not significant).

The competition experiment was conducted in different stress conditions (Figure 4A). Altered growth of cells overexpressing mutant or wild type sequences was detected in LB-based media supplemented with different stressors, whereas plain LB medium did not have a significant influence on the relative growth of mutant and wild type. For instance, addition of the organic acids L-malic acid and malonic acid as stressors led to better growth of cells containing the wild type plasmid compared to cells expressing the mutated sequence, indicated by a significantly lower CI compared to the t_0_ condition; thus, the presence of *pop* is advantageous in these conditions. Addition of the acidic substances resulted in an initial pH shift from 7.4 to 5.8. A higher CI was detected when LB was buffered with bicine to a pH of 8.7. However, LB adjusted to acidic (pH 5.8) or near neutral (pH 7.4) milieu with the biologic buffers MES and MOPS, respectively, did not result in significant growth differences.

We estimated copy number differences of competitors separately grown in LB, LB + L-malic acid and LB + bicine to exclude competitive growth effects occurring due to different plasmid amounts within the cells. In each condition, the cycle threshold differences of the plasmid-encoded gene coding for β-lactamase (*bla*) and the genome-encoded gene coding for the siroheme synthase (*cysG*) in cells overexpressing *pop* or Δ*pop* were determined (ΔCq). The ΔCq ratios of the two competitive strains do not significantly differ before and after growth in any of the conditions (Welch two-sample *t*-test, *p*-values > 0.05, Figure 4B and Supplementary Table S4). Thus, growth differences are true effects due to overexpression of *pop* and Δ*pop*, and not merely copy-number variations of the plasmids.

In accordance with the growth advantage of the wild type in the presence of malic acid (Figure 4A), *pop* RNA quantification with qPCR showed increased mRNA levels of *pop* relative to 16S rRNA levels in the presence of L-malic acid (fold change 2.4, Figure 4C and Supplementary Table S5). In contrast, less mRNA was detected in bicine-buffered LB medium at pH 8.7 (fold change 0.35, Figure 4C). Although significantly different *Cq* values were detected only in the alkaline medium (one-tailed Welch two sample *t*-test, *p*-value = 0.03), we suggest that the fold change in L-malic acid also differs, though the *p*-value is 0.17, as *p*-values are often combined with a fold change to identify differentially expressed genes (e.g., Huggins et al., 2008; McCarthy and Smyth, 2009). We find that *pop* expression is differentially regulated between malic acid and bicine based on these qPCR results (fold change 6.9).

Next, genomic knock-outs for *pop* in *E. coli* O157:H7 EDL933 were constructed (Δ*pop* and Δ*pop* v2). Base substitutions were introduced 64 and 282 bp downstream of the potential start codon CTG. The stop codon mutation of EHEC Δ*pop* v2 was inserted after the codon GTG in peak region 3 identified in ribosome profiling data (Figures 2A–C, also discussed below). The mutations each led to a stop codon in *pop*, whereas amino acids in *ompA* remained unchanged (Figure 1A). We tested the mutant Δ*pop* and Δ*pop* v2 in several relevant stress conditions in competitive growth against the wild type strain, but did not detect a significant difference of growth in any condition for either of the mutants (Supplementary Figure S2).

Based on the clear effect of overexpression, we propose that *pop* codes for a protein, as mRNAs transcribed from the intact sequence and the translationally arrested variant differ in one nucleotide only. Thus, RNA interactions of *pop* and *ompA* are probably not affected. Opposite overexpression phenotypes were found in alkaline buffered and acidified media, so we propose a pH-dependent function. In line with this hypothesis, the mRNA is differentially regulated in various pH conditions (Figure 4C).

### The Transcriptional Unit of *pop* Includes an Active Promoter and a Rho-Independent Terminator

**FIGURE 5:**
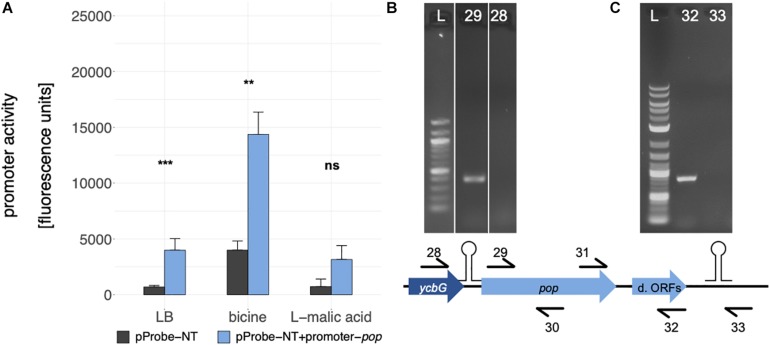
Analysis of the *pop* transcriptional unit. **(A)** Promoter activity assay for the promoter of *pop*, which was introduced in the promoterless GFP vector pProbe-NT. Mean fluorescence units of *E. coli* Top10 cells with the different constructs in culture conditions as indicated are given. Error bars show standard deviations. Statistical significance between empty vector (gray bars) and the promoter construct (blue bars) was tested with a Welch two sample *t*-test (** *p* ≤ 0.01; *** *p* ≤ 0.001; ns, not significant). **(B)** Test for mono- or polycistronic mRNA. An agarose gel of RT-PCRs is shown. RNA was reverse transcribed in cDNA with *pop* specific reverse primer 30. Two different forward primers, binding within *ycbG* (no. 28) or within *pop* (no. 29), were combined with a *pop* reverse primer (no. 30). L: 100 bp DNA Ladder (NEB); 28: PCR with primers 28 + 30; 29: PCR with primers 29 + 30. **(C)** Test for the predicted rho-independent terminator. An agarose gel of RT-PCRs is shown. RNA was reverse transcribed in cDNA with *pop* specific reverse primers 32 or 33 binding upstream (no. 32) or downstream (no. 33) of the stem loop structure. For PCR, these two reverse primers were combined with a *pop* forward primer (no. 31). L, 1 kb plus DNA Ladder (NEB); 32: PCR with primers 32 + 31; 33: PCR with primers 33 + 31; d. ORFs, downstream ORFs.

Cappable-seq (Ettwiller et al., 2016) is a recently developed approach detecting the TSS of mRNA with next generation sequencing. Using this method, a weak but significant transcriptional start site was determined at genome position 1235862 in the intergenic region between *ycbG* and *pop* in independent biological experiments (Figure 1, TSS; Supplementary Figure S1). Two independent bioinformatics tools, BPROM and bTSSfinder, were used to analyze the upstream region of the TSS for potential promoter sequences. Both programs identified a σ^70^ promoter [BPROM LDF score 0.59, Solovyev and Salamov (2011), bTSSfinder score 1.86, Shahmuradov et al. (2017), Figure 1B and Supplementary Figure S1]. Although the distance between the transcriptional start site and −10 box of the promoter is not optimal (2 bp instead of approx. 7 bp), promoter sequence activity was verified by means of a GFP-assay (Figure 5A). We found a significantly enhanced fluorescence in cells harboring the plasmid containing the putative promoter sequence compared to those with the empty vector in LB and bicine-buffered medium, indicating an active promoter sequence upstream of the TSS of *pop*. The fluorescence signal of the promoter in the basic milieu (pH 8.7) was strikingly higher, but this may result from GFP accumulation during longer incubation times necessary in this medium (Miller et al., 2000).

Since the promoter activity for *pop* is weak compared to promoters of annotated genes, we tested for polycistronic expression starting from the promoter of *ycbG*. Reverse transcription PCR (RT-PCR) was performed to examine the transcript of *pop* (Figure 5B). No mRNA spanning both genes was detectable, thus, we propose that *pop* is transcribed from the tested promoter monocistronically.

A 120 bp long rho-independent terminator was predicted 295 bp downstream of the stop codon of *pop* using FindTerm (Solovyev and Salamov, 2011). Hypothetical secondary structures of 30-bp segments of this region were created with the tool Quickfold of Mfold (Zuker, 2003). A stable stem loop structure (Δ*G* = −8.6 kcal/mol) within bases 35–78 of the predicted terminator sequence was detected (Figure 1D). To verify the 3′-end of the mRNA downstream of the hairpin structure, RT-PCRs were performed. We used reverse primers binding either within the downstream ORFs or further downstream, beyond the secondary structure. We observed that *pop* and the downstream ORFs are co-transcribed and transcription is terminated just downstream of the predicted stem loop structure (Figure 5C).

Based on these results, we conclude that *pop* forms an approximately 1120 bp long transcriptional unit covering almost the entire open reading frame of the annotated gene *ompA*, excluding the upstream gene *ycbG* but including the downstream ORFs, ending with a rho-independent terminator.

### Western Blot of Pop

**FIGURE 6:**
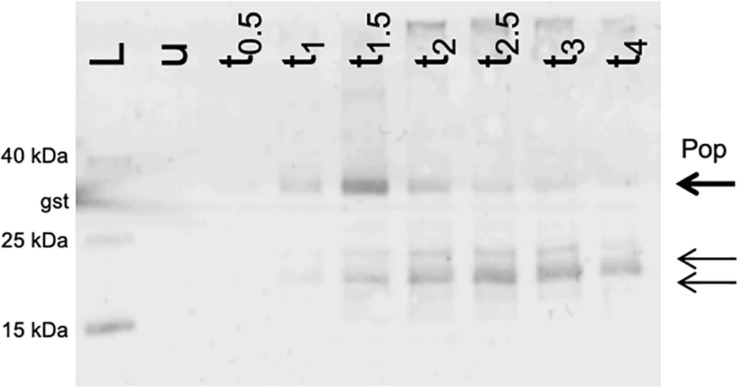
Western blot of Pop protein. Expression of *pop* in frame with a C-terminal SPA-tag. Cells were grown in LB at pH 7.4 and harvested before induction and up to 4 h after induction (t_*x*_) and normalized cell numbers were separated on 16% tricine gels. The arrows indicate the band of the putative full-length protein Pop (bold arrow) and two shorter products (thin arrows). The experiment was conducted in LB at pH 7.4. L, Spectra Multicolor Low Range Protein Ladder (Thermo Scientific) with additional internal Western blot control glutathione-S-transferase (band sizes are indicated); *u*, whole cell extract from uninduced cells; *t*_*x*_, whole cell extract from samples harvested after *x* = 0.5, 1, 1.5, 2, 2.5, 3, and 4 h after induction with arabinose.

Since we detected an active promoter (Figure 5A) and phenotypes in competitive overexpression experiments (Figure 4A), the coding capacity of *pop* was assessed using Western blotting. *pop* was cloned in-frame with an SPA tag (7.7 kDa; following Zuker, 2003) on a pBAD-based plasmid, and overexpressed in EHEC. SPA-tagged proteins were visualized after separating whole cell lysates on tricine gels. The experiment was performed in LB at pH 7.4 (Figure 6). Besides the expected full-length protein (theoretically 30 kDa, detected approx. 34 kDa), shorter products were immunostained (approx. 20 and 24 kDa). The amount of the full Pop protein appears to increase within the first 1.5 h after induction and decreases afterward when overexpressed, pointing to an instability of the protein. However, this experiment does not prove natural occurrence of the protein, due to artificial overexpression of the protein. On the other hand, detection of an endogenously expressed protein in Western blots failed as *pop*-tagged cells could not be recovered due to technical issues. Nevertheless, we could detect an initially stable Pop protein, supporting a protein coding potential of *pop* in general.

### Bioinformatic Evidence for *pop* Being a Protein-Coding Gene

Protein databases were searched for Pop homologs in order to find hints of a specific function. No significant similarities with annotated proteins were found using blastp analysis in PDB (Protein Data Bank), UniProtKB/Swiss-Prot and the Ref-Seq protein database, but homologous proteins were detected in NCBI’s non-redundant protein sequence (nr) database. However, the hits covered at best 67% of the amino acid sequence of *pop*. A deeper analysis of the top hit (uncharacterized protein, 67% coverage, 99% identity, e-value of 4^–91^) and the genomic sequence of the target organism *Shigella sonnei* showed that its *ompA* homolog was not annotated due to ambiguous bases at its 5′ end, which resulted in a missing start codon for *ompA* in this case. Consequently, *pop* was “allowed” to be predicted *ab initio*, as *ompA* had no obvious gene structure and was, therefore, rejected during annotation. This result corroborates the known function of many algorithms like Glimmer, Prodigal or Prokka, which systematically avoid annotation of long (non-trivially) overlapping genes (Delcher et al., 2007; Hyatt et al., 2010; Seemann, 2014). Further, NCBI explicitly forbids long overlaps in their prokaryote genome annotation standards (NCBI, 2018). To check whether *pop* is recognized by gene finding algorithms in the case of an absent *ompA* annotation, we applied Prodigal to four genomes of bacteria in the family *Enterobacteriaceae* (*E. coli* O157:H7 EDL933, *S. dysenteriae*, *K. pneumoniae*, *E. cloacae*). Potential start codons of *ompA* were masked with N bases in each genome and consequently *ompA* was not detected. In contrast, *pop* was predicted as a protein-coding gene in all four genomes (Supplementary Table S6). The absolute prediction scores of all annotated protein-coding genes in this analysis ranged from −0.5 to >1000 in EHEC. The total score of *pop* is 14.37 and falls within the lowest 10% of the 5351 predicted EHEC coding sequences. Nevertheless, sequences with even lower scores than *pop* represent conserved annotated genes, e.g., a fimbrial chaperon or the entericidin A protein, to name two of many. Thus, *pop* has elements of a gene structure which enable its identification as a protein-coding gene when *ompA* is masked. In the normal case, *pop* is apparently rejected in annotation solely due to its overlapping gene partner *ompA* rather than any property of the sequence itself.

## Discussion

Antisense transcription is a widespread phenomenon in bacteria and often connected to regulatory function of the RNAs (Dornenburg et al., 2010; Ettwiller et al., 2016). However, there is increasing evidence that antisense RNAs can be templates for ribosomes to synthesize proteins (Miranda-CasoLuengo et al., 2016; Weaver et al., 2019). So far, characterized non-trivially overlapping genes are typically short (e.g., Fellner et al., 2014; Haycocks and Grainger, 2016; Hücker et al., 2018a). Therefore, the discovery and analysis of *pop* with a length of 200 amino acids is of special interest.

The number of coding sequences in bacteria predicted by genome annotation algorithms is underestimated, in particular because neither small genes nor genes with extensive overlap are considered to be true genes (Burge and Karlin, 1998; Delcher et al., 2007; Hücker et al., 2017). Therefore, it is not surprising that *pop* has until now escaped attention. In our study, we detected translation of *pop* in three pathogenic *E. coli* strains. Although whether ribosome-profiling signals indicate translation of genes in all cases is debated, independent confirmation of expression or function for specific genes can be achieved either by chromosomal tagging (e.g., Baek et al., 2017; Meydan et al., 2019) or functional characterization, as for example presented here using competitive growth. The pattern of translation in ribosome-profiling data of this ORF is conserved across widely divergent *E. coli* strains, albeit with very low translation in some strains. It has been shown that translation of even short proteins in *E. coli* is associated with a significant bioenergetic cost (Lynch and Marinov, 2015), and specific translation of non-functional genes would therefore be expected to be acted against by selective processes relatively quickly. The strains compared diverged more than 4 million years ago according to molecular clock methods in the case of K12 and Sakai (Reid et al., 2000). This corresponds to more than 1 billion generations, and LF82 is still more distantly related. Consequently, we would expect all non-functional translated products shared with the common ancestor of these strains to have been lost. In contrast to conserved translation in pathogenic *E. coli*, *pop* translation was not observed in the well-studied *E. coli* K12. This finding, in combination with the discarding of *pop* in automated annotation, as it is embedded antisense in the conserved outer membrane protein *ompA*, leads us to propose that it was simply overlooked so far.

We studied the transcriptional unit of *pop* and identified (i) a TSS (ii) downstream of a σ^70^ promoter, (iii) a potentially coding ORF (i.e., *pop*) with a putative CTG start codon, and (iv) an experimentally verified rho-independent terminator of *pop*.

In the ribosome profiling data, we identified three peak regions, which are evidence for translation initiation sites in translatome data (Oh et al., 2011; Woolstenhulme et al., 2015); a putative start codon of *pop* could be contained in each of these (regions 1–3 in Figure 2A and Supplementary Figure S1). All regions are covered with a substantial number of ribosomal profiling reads, and region 2 is covered best, particularly in EHEC EDL933. We assume that translation for *pop* starts in region 2, especially since a Shine-Dalgarno motif for ribosome binding was predicted and ribosome profiling data across divergent strains point to a putative translation initiation site therein. As mentioned, a nearby CTG is found downstream of a ribosome-binding site, representing a rare but sometimes-used start codon for prokaryotes (Spiers and Bergquist, 1992; Sussman et al., 1996; Hecht et al., 2017; Yamamoto et al., 2018).

Furthermore, a TTG start codon is present in region 1, representing the longest potential ORF for *pop*. However, we could not find evidence for a TSS or SD-sequence, though the latter is not obligatory for gene expression (Moll et al., 2002; Gualerzi and Pon, 2015). This TTG was the start codon predicted by Prodigal as the most probable one, however, as the upstream gene *ycbG* has a predicted terminator (ΔG = −12.20 kcal/mol, indicated in Figures 1, 5B) and bicistronic expression of *pop* along with *ycbG* was excluded by our data, we propose that this TTG is not a start codon here.

The start codon in region 3 (GTG) is located 45 amino acids downstream of the mutation introduced in *pop* for analysis in competitive growth. We did not find a phenotype for a translationally arrested mutant regarding this putative start codon. Furthermore, overexpression growth phenotypes found in competitive growth experiments are not conferred by the protein translated from this start codon. While these points are strong evidence that this GTG is not the start codon, formation of a protein isoform not carrying a phenotype in the conditions analyzed here cannot be excluded.

In addition to the gene structure, the Pop protein was analyzed in our study. A Western blot verified the presence of a protein, which appears to be unstable when expressed from the plasmid. Nevertheless, native protein expression could not be investigated immunologically and natural occurrence of the protein Pop remains unclear. Pop might be stable, degraded or exist in different isoforms, a phenomenon reported for some bacterial proteins recently (Waters et al., 2011; Di Martino et al., 2016; Nakahigashi et al., 2016; Vanderhaeghen et al., 2018; Meydan et al., 2019).

Most importantly, competitive overexpression growth assays conducted in this study are the best indication for a proteinaceous nature of the *pop* gene product. As recently shown, not only loss-of-function screenings but also overexpression phenotyping is an appropriate approach to find novel genes and to elucidate their function (Mutalik et al., 2019). However, as shown previously, overexpression of unnecessary but usually non-toxic proteins often leads to decreased growth rates (Dong et al., 1995; Shachrai et al., 2010). This could be assumed in our assay conducted in bicine-buffered LB, in which cells expressing the full-length protein had significantly lower growth. Nevertheless, as growth behaviors of mutant and wild type *pop* expressing cells did not change in pure LB, the phenotype seems to be rather specific for the alkaline stress conditions and not due to an effect of overexpression stress. However, in acidified medium the cells had a growth advantage in comparison to cells expressing the truncated form and, thus, *pop* overexpression is beneficial to EHEC at low pH. This is important since this effect cannot be explained by stressed cells due to protein overexpression. In contrast, analysis of a genomic knock-out suggests that the absence of the protein is not deleterious for EHEC under the conditions tested. While it has been shown that effects of overexpression and knock-out can be complementary, this is not always the case (Prelich, 2012). Several examples exist in which the actions of genes can be compensated by each other [e.g., CLN1 and CLN2 in *S. cerevisiae*, Hadwiger et al. (1989); cold shock proteins in bacteria, Xia et al. (2001)]. For CLN1 and CLN2, both have similar effects when overexpressed separately, but absence of one of the genes can be balanced out by the other and only a double knock-out has a phenotype.

In summary, we suggest that the investigated open reading frame encodes a protein, since it has all structural features of a protein coding gene, is translated and it shows overexpression phenotypes in pH stress. We propose the name *pop* (pH-regulated overlapping protein-coding gene) for this novel overlapping gene. It should be noted that the *hemC/F/H/L* genes were previously referred to as *popA/B/C/E* but the OLG *pop* is not associated with any function of these. It could be speculated that the positive effect of overexpressed *pop* in acidic medium correlates with the acid tolerance of EHEC necessary to overcome the acidic barrier in the stomach after ingestion (Nguyen and Sperandio, 2012). If true, *pop* could be a pathogenicity or host-environment related gene of EHEC only activated upon specific stress.

Long ORFs embedded antisense to annotated genes like *pop*, as well as other overlapping ORFs, may form a hitherto greatly underestimated source of proteins. Recently developed methods like dRNA-seq (Sharma et al., 2010) and Cappable-seq (Ettwiller et al., 2016) identified hundreds of TSSs antisense to annotated genes producing antisense transcripts with unknown translation status and function. Modern ribosome profiling techniques, including stalling ribosomes at translation initiation sites, identified several unambiguous start codons for protein coding genes, which overlap with annotated genes either in sense or in antisense direction (Meydan et al., 2019; Weaver et al., 2019). We suggest that these “abnormal” transcriptional and translational signals in next generation sequencing analysis should not be neglected but analyzed in more detail, as has been conducted for the long overlapping gene *pop.* Many novel functional elements, especially for pathogenicity in novel hosts or survival in new niches, might be “hiding” in the genome of any bacterium.

## Data Availability Statement

The datasets generated for *E. coli* LF82 can be found in the Sequence read archive; accession numbers are SRR11217090, SRR11217089, SRR11217088, and SRR11217087.

## Author Contributions

BZ performed the experimental analysis on *pop* in EHEC EDL933 and database searches. ZA conducted the analysis of ribosomal profiling data. MK performed the ribosomal profiling in LF82. SS and KN supervised the study. BZ wrote the first draft of the manuscript including the figures with the help of KN and SS. All authors read and approved the final version of the manuscript.

## Conflict of Interest

The authors declare that the research was conducted in the absence of any commercial or financial relationships that could be construed as a potential conflict of interest.
