# Supplementary material for: A Novel pH-Regulated, Unusual 603 bp Overlapping Protein Coding Gene pop Is Encoded Antisense to ompA in Escherichia coli O157:H7 (EHEC)
Source: Front Microbiol. 2020 Mar 20;11:377. doi: 10.3389/fmicb.2020.00377 (PMC7103648; doi:10.3389/fmicb.2020.00377)
Supplement: Supplementary file 1 [file Data_Sheet_1.docx]

Supplementary Material

# Supplementary Figures and Tables

## Supplementary Figures

**
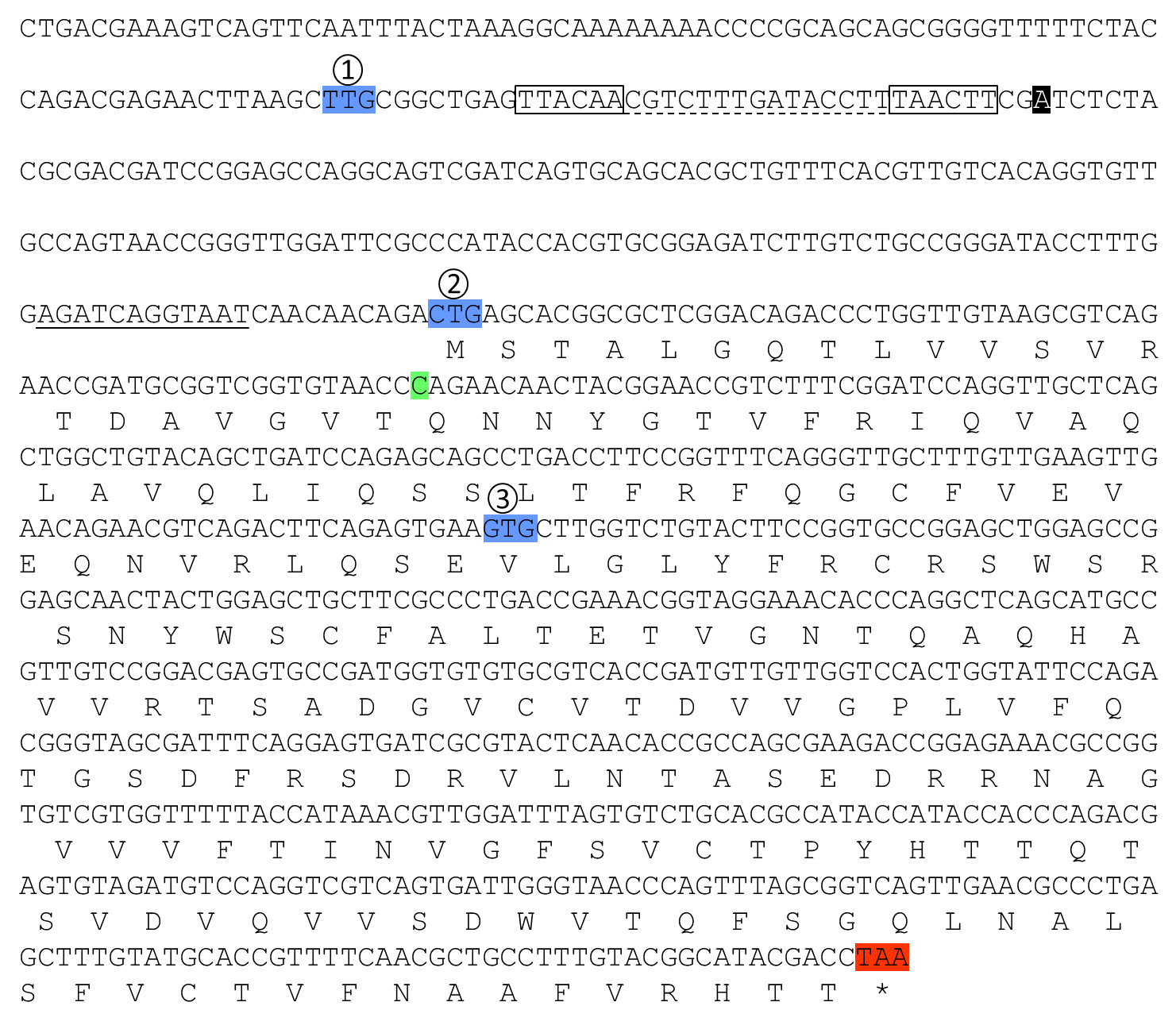
**

**Supplementary Figure S1** Genomic sequence of *pop*. Nucleic acid and proposed amino acid sequence of *pop* are shown. Several features are highlighted: black, TSS; blue, possible NTG start codons (1, 2, 3); red, stop codon (*); green, mutated position (C → T) resulting in a premature stop codon in *pop*. Black boxes and dashed line indicate the predicted promoter with -35 and -10 box and spacer region, respectively. The Shine-Dalgarno sequence upstream of start codon 2 is underlined.

**
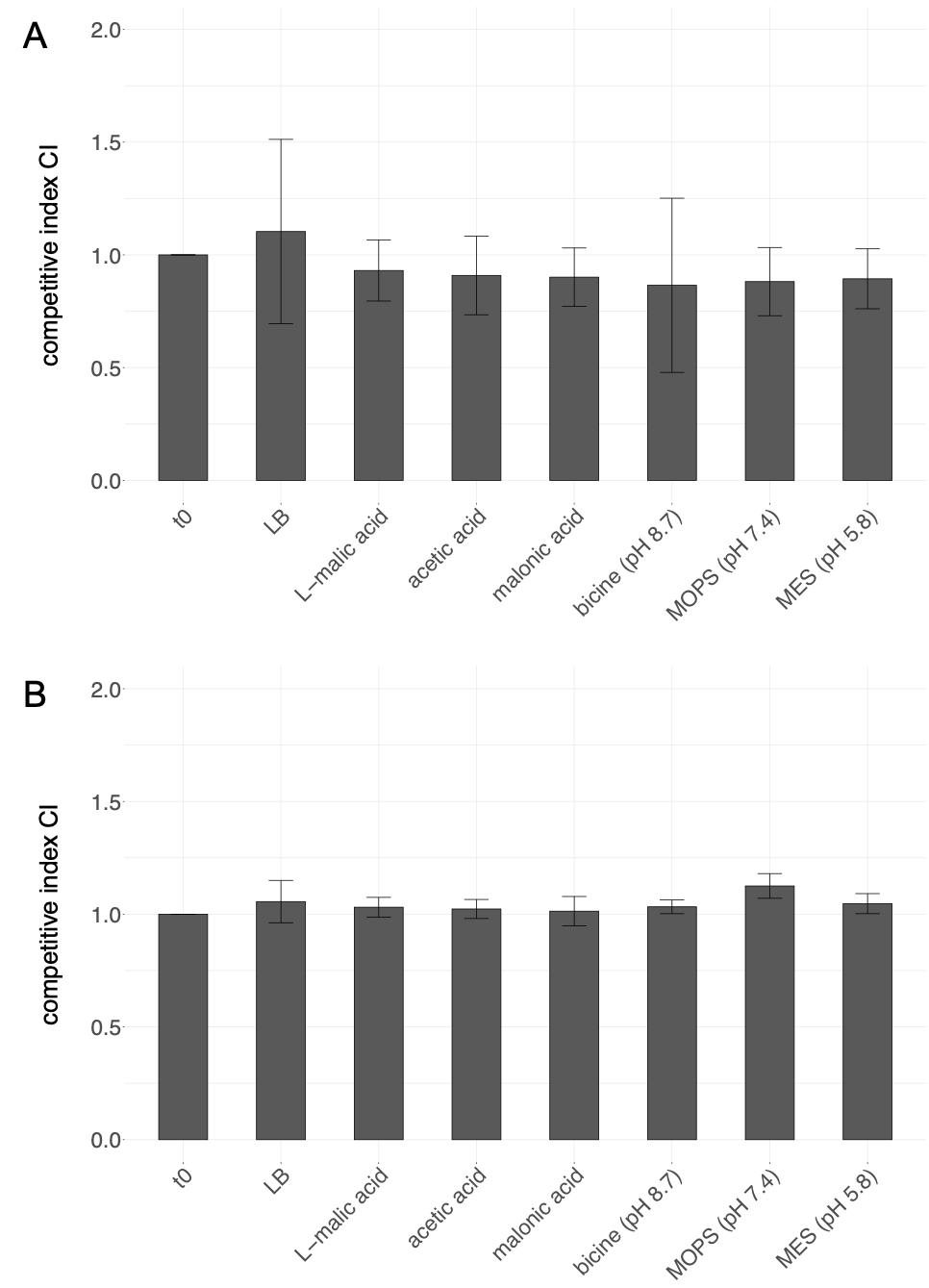
**

**Supplementary Figure S2** Competitive growth using genomic knock-out mutants (A) EHEC ∆*pop* and EHEC ∆*pop* v2. The genomic knock-outs were grown together with EHEC wild type in plain LB or indicated stress conditions (LB medium either supplemented with organic acids or biological buffers). Mean competitive indices CI are given as the ratio of relative abundance of cells expressing mutant or wild type plasmid measured by peak heights, i.e. fluorescence intensities in sequencing electropherograms at the mutated positions, in different culture conditions regarding the input ratio t_0_. Error bars indicate standard deviations.

## Supplementary Tables

**Supplementary Table S1** Primers, bacterial strains and plasmids used in this study.

**Supplementary Table S2** Cultivation stressors, antibiotics and media supplements with corresponding concentrations used in this study.

**Supplementary Table S3** Evaluation of ribosome profiling and RNAseq. Chromosome coordinates (E, F, G and N, O, P) of the open reading frames (H and Q) considered in this study. For each strain (A, B; EDL933, Sakai, LF82, MG1655) in the analyzed ribosomal profiling (C, D) and RNAseq (L, M) datasets, read counts (I, R), RPKM (J, S), and coverage (K, T) are given. Column U, RCV values (i.e. RPKM ribosome profiling / RPKM RNAseq)

**Supplementary Table S4** qPCR for plamid copy number estimation. Quantification cycles (Cq) of genomic and plasmid DNA before and after growth of EHEC overexpressing either *pop* (pBAD+*pop*) or Δ*pop* (pBAD+Δ*pop*) in LB, LB + L-malic acid (4 mM) and LB + bicine (100 mM, pH 8.5) for three technical replicates for each of three biological replicates are given. Averaged Cq values of plasmid DNA (Cq(*bla*)) were normalized to averaged Cq values of genomic DNA (Cq(*cysG*)) to estimate the copy number of the pBAD plasmids. The ratios of copy numbers were calculated. Pure genomic DNA and a plasmid preparation carried out with a commercially available isolation kit (GenElute Plasmid Miniprep Kit, Sigma-Aldrich) were used as controls. NTC: non template control. Missing detection of a PCR product via fluorescence is indicated with NA (‘not available’).

**Supplementary Table S5** Quantification cycles (Cq) of *pop* mRNA (green) and 16S rRNA (blue) expression after EHEC growth in LB, LB + L-malic acid (4 mM) and LB + bicine (100 mM, pH 8.5) for three technical replicates for each of three biological replicates. Averaged Cq values of *pop* were normalized to averaged Cq values of 16S rRNA (ΔCq) and the average fold change between two conditions was calculated with 2^-ΔΔCq^. No-RT controls were measured for each RNA sample once. Missing detection of a PCR product via fluorescence is indicated with NA (‘not available’).

**Supplementary Table S6** *pop* prediction with Prodigal. For each species (A, EHEC EDL933, *Shigella dysenteriae*, *Klebsiella pneumoniae*, *Enterobacter cloacae*) chromosome coordinates (B, C, D) of identified open reading frames are given. The best hit is shaded grey, respectively. Total score (E), coding potential (F), start score (G), start codon (H), RBS motif (I), spacer (J), RBS score (K), upstream score (L), type score (M), and GC concent of the ORF (N) are listed.

## Original gel images and blots

to Figure 5B:


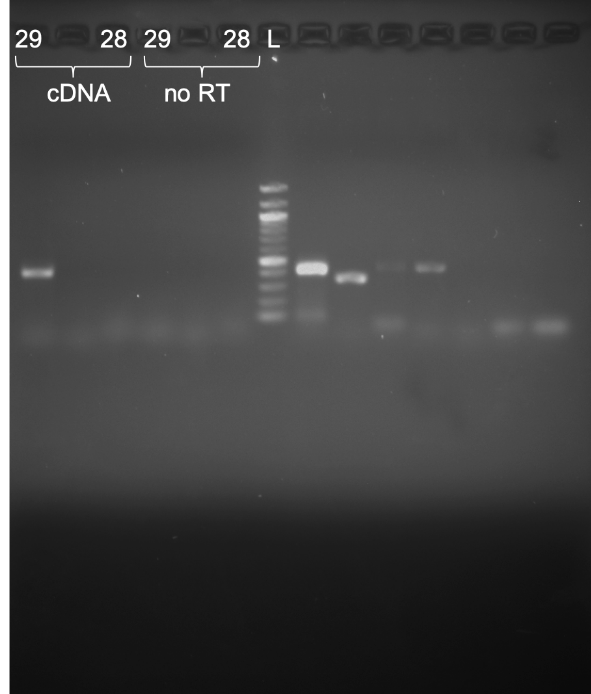
relevant lines marked with L, 29 or 28

to Figure 5C:
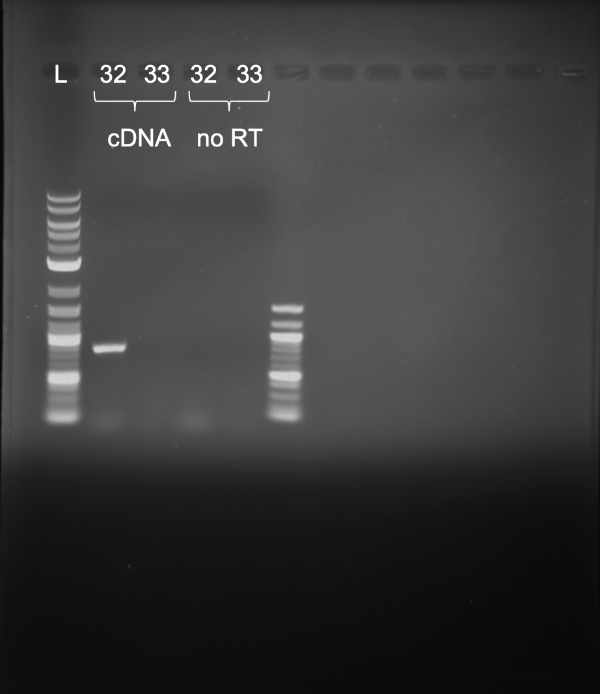


relevant lines marked L, 32 or 33


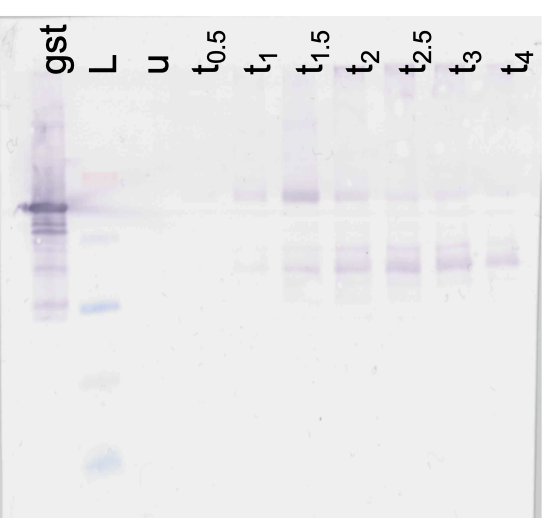


Additional lane gst: control protein glutathione S-transferase (gst)
